# Risk Factors of Periprocedural Bradycardia during Primary Percutaneous Coronary Intervention in Patients with Acute ST-Elevation Myocardial Infarction

**DOI:** 10.1155/2019/4184702

**Published:** 2019-11-15

**Authors:** Yong Li, Shuzheng Lyu

**Affiliations:** ^1^Emergency and Critical Care Center, Beijing Anzhen Hospital, Capital Medical University Beijing Institute of Heart Lung and Blood Vessel Diseases, Beijing 100029, China; ^2^Department of Cardiology, Beijing Anzhen Hospital, Capital Medical University Beijing Institute of Heart Lung and Blood Vessel Diseases, Beijing 100029, China

## Abstract

**Background:**

Evidence available suggests that periprocedural bradycardia negates the benefit of primary percutaneous coronary intervention (PPCI) and worsens the prognosis of patients with acute ST-elevation myocardial infarction (STEMI).

**Objective:**

To investigate the risk factors of periprocedural bradycardia during PPCI in patients with acute STEMI.

**Methods:**

We enrolled 2,536 acute STEMI patients who had PPCI from November 2007 to June 2018 in Beijing Anzhen Hospital, Capital Medical University. We divided all patients into two groups according to periprocedural bradycardia (preoperative heart rate ≥50 times/min, intraoperative heart rate <50 times/min persistent or transient) during PPCI: periprocedural bradycardia group (434 cases) and control group (2102 cases). We compared demographic, clinical, and angiographic characteristics of the two groups. We analyzed the risk factors of periprocedural bradycardia.

**Results:**

The incident rate was 17.1% (434/2536). Logistic regression analysis showed that the differences between the two groups in no-reflow, the culprit vessel was LAD, using thrombus aspiration devices during operation, gender, completely block of culprit vessel, and intraoperative hypotension were statistically significant (*P* < 0.05). The area under the receiver operating characteristic curve was 0.8390.

**Conclusions:**

No-reflow, the culprit vessel was not LAD, using thrombus aspiration devices during operation, gender, completely block of culprit vessel, and intraoperative hypotension may be independent risk factors for predicting periprocedural bradycardia during PPCI in patients with acute STEMI. We registered this study with WHO International Clinical Trials Registry Platform (ICTRP) (registration number: ChiCTR1900023214; registered date: 16 May 2019).

## 1. Background

All types of conduction disturbances can occur in the context of an acute myocardial infarction (AMI) [1]. Hemodynamic compromise secondary to significant bradycardia can have deleterious effects on organ perfusion, which can complicate recovery and negatively impact survival (through renal, hepatic, or cerebral ischemia) [1]. Primary percutaneous coronary intervention (PPCI) is the best available reperfusion strategy for acute ST-elevation myocardial infarction (STEMI). Prevention of periprocedural bradycardia is a crucial step in improving prognosis of patients with STEMI. There exists a need for tools that will be able to aid early identification of patients at increased risk of periprocedural bradycardia. This may enable patients at a heightened risk of periprocedural bradycardia to be treated with the most appropriate individualised treatment early. We want to investigate the risk factors of periprocedural bradycardia.

## 2. Methods

### 2.1. Source of Data

The derivation cohort was 2,536 patients with acute STEMI presenting within 12 hours from the symptom onset who were consecutively treated with PPCI in Emergency and Critical Care Center, Beijing Anzhen Hospital, Capital Medical University, from November 2007 to June 2018.

#### 2.1.1. Inclusion Criteria

The inclusion criteria are as follows: (1) patients with acute STEMI presenting within 12 hours from the symptom onset who were treated with PPCI. (2) Age above 18 years and less than 80 years old male and nonpregnant women. The term AMI should be used when there is acute myocardial injury with clinical evidence of acute myocardial ischemia and with detection of a rise and/or fall of cTnI values with at least one value above the 99th percentile upper reference limit and at least one of the following: (1) symptoms of myocardial ischemia and (2) new ischemic ECG changes [2]. The diagnosis of STEMI was established in the presence of chest pain lasting for >20 minutes associated with electrocardiographic changes (ST-segment elevation of >1 mm in at least 2 extremity electrocardiographic leads or >2 mm in at least 2 contiguous precordial leads). The diagnosis was confirmed by coronary angiography in all patients. (3) Patients who had taken beta-blockers on admission or not were included. (4) Patients who had the initiation of catecholamine before the procedure or not were included.

#### 2.1.2. Exclusion Criteria

The exclusion criteria are as follows: (1) patients who received thrombolysis; (2) patients who received bivalirudin; (3) cardiac rupture, ventricular septal perforation, and other mechanical complications; (4) pulmonary embolism, aortic dissection, and acute cerebrovascular disease.

Prior to emergency angiography, all patients received 300 mg of aspirin, 300 to 600 mg of clopidogrel, or 180 mg of ticagrelor and unfractionated heparin (patients who received bivalirudin were excluded).

### 2.2. Evaluation and Diagnosis of Periprocedural Bradycardia

Periprocedural bradycardia means preoperative heart rate was ≥50 times/min, and intraoperative heart rate was <50 times/min persistent or transient [1]. Preoperative heart rate was based on the medical record; intraoperative heart rate was based on the operation record.

### 2.3. Predictors

We selected 10 predictor variables for inclusion in our prediction rule from the larger set according to clinical relevance and the results of baseline descriptive statistics in our cohort of patients treated with PPCI. 10 potential candidate variables were age, gender, hypertension history, diabetes history, coronary artery disease history, culprit vessel site, completely block of culprit vessel, no-reflow, intraoperative hypotension (preoperative blood pressure was ≥90/60 mmHg and intraoperative blood pressure was <90/60 mmHg persistent or transient), and using thrombus aspiration devices during operation.

Angiographic criteria were used for the diagnosis of no-reflow [3]. No-reflow was defined as Thrombolysis In Myocardial Infarction risk score (TIMI) < III [4]. Preoperative blood pressure, age, gender, hypertension history, diabetes history, and coronary artery disease history were based on the medical record. No-reflow, culprit vessel site, completely block of culprit vessel, using thrombus aspiration devices during operation, and intraoperative hypotension were based on the operation record.

### 2.4. Statistical Analysis

We presented data as mean ± SD or *n* (%). We kept all continuous data as continuous and retained on the original scale. We used univariable and multivariable logistic regression models to identify the correlates of periprocedural bradycardia during PPCI. We entered all variables of Table 1 into the univariable logistic regression. We constructed a multivariable logistic regression model using the backward variable selection method based on the variables that resulted significant from univariable logistic regression.

Discrimination was the ability of the model to differentiate between patients who do and do not experience periprocedural bradycardia during the study period. This measure was quantified by calculating the area under the receiver operating characteristic (ROC) curve (AUC).

We performed statistical analyses with STATA version 15.1 (StataCorp, College Station, TX). All tests were two-sided, and a *P* value <0.05 was considered statistically significant.

## 3. Results

### 3.1. Participants and Predictors of Periprocedural Bradycardia

During PPCI procedure, 434 patients had periprocedural bradycardia (periprocedural bradycardia group), and 2102 patients had no periprocedural bradycardia (control group). The results are shown in Table 1.

### 3.2. Predictors of Periprocedural Bradycardia

We used univariable and multivariable logistic regression to identify predictors of periprocedural bradycardia during PPCI. We identified 8 variables (age, gender, the culprit vessel was left anterior descending (LAD), the culprit vessel was right coronary artery (RCA), completely block of culprit vessel, using thrombus aspiration devices during operation, no-reflow, and intraoperative hypotension) as predictors of periprocedural bradycardia in univariable analysis. After application of the backward variable selection method, 6 variables (no-reflow, the culprit vessel was not LAD, using thrombus aspiration devices during operation, gender, completely block of culprit vessel, and intraoperative hypotension) remained as significant independent predictors of periprocedural bradycardia during PPCI. The results are shown in Tables 2 and 3.

According to the above risk factors, we can calculate the predicted probability of periprocedural bradycardia using the following formula: *P*=1/(1 + exp(−(−1.937454 + −0.6133868 ∗ G + 0.8055676 ∗ CBCV + 0.6712826 ∗ CNR + 0.556152 ∗ TA + −1.932014 ∗ LAD + 2.308423 ∗ IH))). LAD = the culprit vessel was LAD, CNR = no-reflow, G = gender, CADH = coronary artery disease history, CBCV = completely block of culprit vessel, IH = intraoperative hypotension, TA = using thrombus aspiration devices during operation, 0 = No, and 1 = Yes; women are coded as 0 and men as 1.

**Figure 1:**
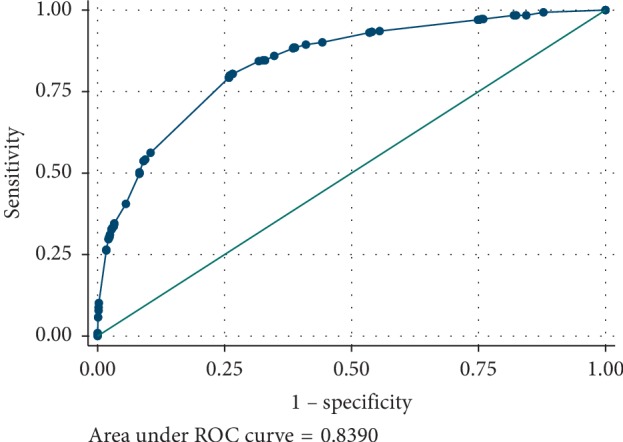
Receiver operating characteristics curve in identifying patients with periprocedural bradycardia.

We drew the receiver operating characteristic (ROC) curve (Figure 1). The area under the receiver operating characteristic curve (AUC) was 0.8390 ± 0.0104 and 95% CI = 0.81859∼0.85943.

## 4. Discussion

All types of conduction disturbances can occur in the context of an AMI, and these are influenced by multiple mechanisms (often concomitant) including ischemia, extent and location of myocardial infarction, reperfusion, and autonomic effects affecting electrical conduction or the sinus or atrioventricular node [1]. We investigated risk factors of periprocedural bradycardia in patients with acute STEMI undergoing PPCI. A frequency of periprocedural bradycardia was 17.1% (434/2536). No-reflow, the culprit vessel was not LAD, using thrombus aspiration devices during operation, gender, completely block of culprit vessel, and intraoperative hypotension are independent risk factors predicting periprocedural bradycardia during PPCI in patients with acute STEMI.

The culprit vessel is RCA or left circumflex coronary artery (LCX) is more likely to evoke periprocedural bradycardia. On the one hand, RCA or LCX supply blood to the sinus node and atrioventricular node. Ischemia reperfusion can induce the apoptosis of sinoatrial node cells [5, 6], so bradycardia was more likely to occur when RCA or LCX is injured. On the other hand, RCA or LCX supplies blood to inferior wall which is the preferential distribution of vagal nerve. Myocardial reperfusion can evoke excitation of cardiac vagal nerve endings and activation of periprocedural bradycardia [7]. Excessive vagus nerve excitation is an important factor that may cause periprocedural bradycardia.

Intraoperative hypotension is often accompanied with periprocedural bradycardia. Acute inferior myocardial infarction often induces transient sinus bradycardia through vagal enhancement, known as Bezold–Jarisch reflex, which is explained by preferential distribution of the vagal nerve in the inferior wall [8]. The Bezold–Jarisch reflex connotes the reflex as described by Dawes: bradycardia, vasodilation, and hypotension [9].

Periprocedural bradycardia is associated with epicardial reperfusion but may also be a sign of no-reflow on coronary angiography. Myocardial reperfusion can evoke excitation of cardiac vagal nerve endings [7], which can cause coronary artery spasm and elicit no-reflow. The vascular endothelium is a multifunctional organ whose integrity is essential to normal vascular physiology. In humans, acetylcholine, the neurotransmitter of the parasympathetic nervous system, which is an endothelium-dependent vasodilator by virtue of the release of nitric oxide or closely related substances, induces coronary dilation in young healthy subjects but causes vasoconstriction in patients with atherosclerosis [10–14]. Acetylcholine is a vascular expansion agent when the endothelial cell function is normal. The expansion effect is mainly because it can induce endothelial cells to secrete nitric oxide [14]. However, smooth muscle cells can induce contraction of vascular, which causes coronary artery spasm when vascular endothelial cells were lost or dysfunction [15, 16].

Using thrombus aspiration devices during operation is more likely to evoke periprocedural bradycardia. Periprocedural bradycardia associated with mechanical thrombectomy using the AngioJet device is a well-described phenomenon in the coronary vessels [17, 18]. Glycopyrrolate, a synthetic anticholinergic agent with a short half-life affecting vagal afferents, has also been used to prevent and treat periprocedural bradycardia associated with the AngioJet device [19, 20]. Periprocedural bradycardia associated with manual thrombus aspiration devices is seldom described in the coronary vessels in the literature. But it does not mean rare.

### 4.1. Study Limitations

Some patients were enrolled >10 years ago; thus, their treatment may not conform to current standards and techniques.

## 5. Conclusions

No-reflow, the culprit vessel was not LAD, using thrombus aspiration devices during operation, gender, completely block of culprit vessel, and intraoperative hypotension may be independent risk factors for predicting periprocedural bradycardia during PPCI in patients with acute STEMI.

## Figures and Tables

**Table 1:** Demographic, clinical, and angiographic characteristics of patients with periprocedural bradycardia and control group during PPCI.

Characteristic [lower limit, upper limit]	Periprocedural bradycardia group (*n* = 434)	Control group (*n* = 2102)	*P* value
Age, years [23, 80]	58 ± 11	57 ± 11	0.004
Man, *n* (%), 0 = no, 1 = yes	338 (77.9)	1775 (84.4)	0.001
Coronary artery disease history, *n* (%), 0 = no, 1 = yes	220 (50.7)	1029 (49)	0.510
Hypertension history, *n* (%), 0 = no, 1 = yes	225 (51.8)	1101 (52.4)	0.839
Diabetes history, *n* (%), 0 = no, 1 = yes	101 (23.3)	551 (26.2)	0.202
Culprit vessel site			
Left anterior descending, *n* (%), 0 = no, 1 = yes	62 (14.3)	1148 (54.6)	<0.001
Left circumflex coronary artery, *n* (%), 0 = no, 1 = yes	59 (13.6)	244 (11.6)	0.246
Right coronary artery, *n* (%), 0 = no, 1 = yes	313 (72.1)	712 (33.9)	<0.001
Completely block of culprit vessel, *n* (%), 0 = no, 1 = yes	384 (88.5)	1357 (64.6)	<0.001
Using thrombus aspiration devices during operation, *n* (%), 0 = no, 1 = yes	343 (79)	1217 (57.9)	<0.001
No-reflow, *n* (%), 0 = no, 1 = yes	82 (18.9)	185 (8.8)	<0.001
Intraoperative hypotension, *n* (%), 0 = no, 1 = yes	159 (36.6)	85 (4)	<0.001

**Table 2:** Predictor of periprocedural bradycardia obtained from multivariable logistic regression models (odds ratio).

Periprocedural bradycardia	Odds ratio	Std. Err.	*Z*	*P* > |*Z* |	95% CI
Gender	0.5415137	0.0822168	−4.04	<0.001	0.4021365∼0.7291979
The culprit vessel was LAD	0.1448561	0.0226479	−12.36	<0.001	0.1066236∼0.1967979
Completely block of culprit vessel	2.237966	0.4119337	4.38	<0.001	1.560186∼3.210191
Using thrombus aspiration devices during operation	1.743949	0.2734684	3.55	<0.001	1.282499∼2.371431
No-reflow	1.956745	0.3402504	3.86	<0.001	1.391626∼2.751353
Intraoperative hypotension	10.05855	1.655183	14.03	<0.001	7.285617∼13.88688
_cons	0.1440703	0.029329	−9.52	<0.001	0.0966701∼0.2147124

**Table 3:** Predictor of periprocedural bradycardia obtained from multivariable logistic regression models (coef).

Periprocedural bradycardia	Coef	Std. Err.	*Z*	*P* > |*Z*|	95% CI
Gender	−0.6133868	0.1518277	−4.04	<0.001	−0.9109636∼−0.3158101
The culprit vessel was LAD	−1.932014	0.1563478	−12.36	<0.001	−2.238451∼−1.625578
Completely block of culprit vessel	0.8055676	0.1840661	4.38	<0.001	0.4448048∼1.166331
Using thrombus aspiration devices during operation	0.556152	0.1568099	3.55	<0.001	0.2488102∼0.8634937
No-reflow	0.6712826	0.1738859	3.86	<0.001	0.3304726∼1.012093
Intraoperative hypotension	2.308423	0.1645547	14.03	<0.001	1.985902∼2.630945
_cons	−1.937454	0.203574	−9.52	<0.001	−2.336451∼−1.538456

## Data Availability

The data used to support the findings of this study are included within the supplementary information file.

## Abbreviations

PPCI: Primary percutaneous coronary intervention
STEMI: ST-elevation myocardial infarction
ROC: Receiver operating characteristic
AUC: Area under the receiver operating characteristic curve
AMI: Acute myocardial infarction
TIMI: Thrombolysis In Myocardial Infarction risk score
RCA: Right coronary artery
LCX: Left circumflex coronary artery
LAD: Left anterior descending.

## Supplementary Materials

Supplementary Materials The data are demographic, clinical, and angiographic characteristics of patients during PPCI. PB = periprocedural bradycardia; CNR = coronary artery no-reflow phenomenon; AGE = age; G = gender; HH = hypertension history; DH = diabetes history; CADH = coronary heart disease history culprit vessel site; LAD = left anterior descending; LCX = left circumflex coronary artery; RCA = right coronary artery; CBCV = completely block of culprit vessel; IH = intraoperative hypotension; TA = using thrombus aspiration devices during operation.
